# The Effects of the Expansion of Dental Care Coverage for the Elderly

**DOI:** 10.3390/healthcare12191949

**Published:** 2024-09-29

**Authors:** Yang Zhao, Beomsoo Kim

**Affiliations:** 1 School of Economics and Management, Yanshan University, Qinhuangdao 066004, China; zhaoyang66@ysu.edu.cn; 2 Department of Economics, Korea University, Seoul 02841, Republic of Korea

**Keywords:** dental insurance coverage expansion, elderly, dental service utilization, imputation-based method, policy evaluation

## Abstract

Background: Expanding dental care coverage for the elderly is globally recommended but not widely implemented due to its high costs and intangible benefits. Methods: This study examined the impact of such an expansion in Korea using the imputation-based method proposed by Borusyak et al. We analyzed data from the Korea National Health and Nutrition Examination Survey (2007–2019) on dental service utilization and chewing ability among older adults. Results: The policy resulted in a 13.5% increase in partial denture use and a 60.5% increase in dental implants among those aged 65 and above. These changes corresponded with reductions in severe chewing difficulty by 23.3% and 13.0%, respectively. No significant changes were observed in full denture use. The price elasticity of demand for partial dentures and dental implants was estimated to be −0.19 and −0.86, respectively. Conclusions: These findings underscore the critical role of affordability in enhancing the utilization of dental implants among the elderly and highlight the importance of appropriately expanding dental insurance coverage to improve oral health outcomes in this population.

## 1. Introduction

Population aging is an irreversible trend worldwide. In 2021, 1 in 10 people worldwide were aged 65 or above. By 2050, this age group is projected to account for 1 in 6 people globally [1]. Tooth loss is a prevalent issue among the elderly. It is estimated that the global average prevalence of complete tooth loss is almost 23% among people aged 60 years and above [2]. The impact of tooth loss on nutritional intake and oral functions (such as chewing, speech, swallowing, smiling, and social interactions) greatly impairs the quality of life and overall health of the elderly [3]. An increasing number of studies has shown numerous associations between oral and systemic diseases that are prevalent in older adults, such as diabetes, cardiovascular disease, dementia, and respiratory diseases [4,5].

Historically, dental health has often been overlooked by policymakers, considered a low-priority issue, and deemed the responsibility of individuals rather than society. This neglect has led to substantial inequalities in dental insurance provision across countries, even among high-income nations, such as the European Union and Canada. For instance, in Canada, up to 40% of lower-income individuals forgo necessary dental care due to cost [6]. Similarly, in countries like Spain, Italy, and Portugal, dental services are primarily offered by the private sector despite the existence of universal health insurance systems [7,8,9].

However, recently, there has been a global shift toward integrating oral health into universal health coverage (UHC) programs. The World Health Organization (WHO) has emphasized the importance of oral health in its global initiatives, yet progress remains limited. A WHO survey from 2017 to 2018 revealed that only 20% of the 101 surveyed countries had oral health programs specifically for older adults, with the figure dropping to 4.8% in low-income countries [10]. This gap is evident in places like China, where a 2022 community survey found that 76% of the elderly respondents suffered from dental diseases, with over half unable to seek care due to financial constraints [11].

Research suggests that dental health can significantly affect other health conditions through its impact on nutritional intake [12,13,14,15]. Severe tooth loss resulting in a lack of functional dentition or edentulism affects nutritional intake and increases the risk of nutritional deficiency in older adults by 21% [5]. Despite this important link, policy evaluations in dental health remain limited, as dental care expansions have been implemented in only a few countries. For instance, in Japan, the co-payment rate for dentures for seniors aged 70 and above was reduced from 30% to 10% for middle-income individuals in 2014 [16]. In the United States, the Affordable Care Act (ACA) enabled states to use federal funding to expand dental access for low-income adults through Medicaid, although the extent of coverage varies by state [17]. More recently, Canada introduced the Canadian Dental Care Plan (CDCP) in 2022 to expand dental insurance coverage [18].

Previous studies exploring the impact of these policy changes have produced mixed results. For example, Ando and Takaku (2016) found that the usage rate of dentures among elderly individuals aged 70 and above in Japan increased by 13 percentage points, but there was no significant improvement in their self-reported chewing ability [16]. In the United States, studies have shown that as Medicaid adult dental benefits have expanded, the utilization of dental services has increased accordingly in each state [19]. States that have expanded Medicaid have experienced an 11.4 percentage point increase in dental visits, a 16.8 percentage point reduction in the prevalence of untreated caries, and an 8.7 percentage point reduction in the prevalence of functional dental problems compared to states that have not expanded Medicaid [20]. Canada’s policy change has not yet been evaluated in academic studies, as it is quite recent.

Korea has also made notable strides in addressing dental health for the elderly by expanding dental coverage not only for specific populations like low-income individuals but for all elderly citizens. Beginning in 2012, the Korean government included dental insurance for full dentures for those over 75, gradually reducing out-of-pocket expenses and extending coverage to individuals aged 65 and older by 2016 [21]. Dental implants were also included in the national health insurance scheme for older adults, although coverage is limited to two implants per person [13]. This provides a unique opportunity to examine the effects of coverage expansion in Korea.

However, studies evaluating policy expansions in Korea have also reported mixed results. For instance, Kim and Kawachi (2020) did not find significant changes in denture utilization, partly due to methodological flaws [22]. Additionally, Choi and Jung (2020) misclassified individuals aged 65 and over as the treated group in 2015, even though coverage only began in 2016 [23]. Furthermore, both studies failed to separately analyze the upper and lower jaws, which could have impacted their findings.

This paper employs the imputation-based estimation method proposed by Borusyak et al. (2024) to determine the causal effects of changes in dental insurance policy on the utilization of dental services and chewing ability among older adults in Korea [24]. This research contributes to the literature in the following ways: it focuses on older adults prone to tooth loss, emphasizes the importance of dental prosthetic services in enhancing oral functions, and utilizes objective data from dental examinations. Additionally, it analyzes the upper and lower jaws as separate samples, providing a more nuanced understanding of dental health needs.

## 2. Materials and Methods

### 2.1. Dental Insurance Policy for the Elderly in Korea

Although the National Health Insurance (NHI) covers all citizens, the dental service coverage by the NHI was limited to conservative treatment, endodontic treatment, extraction, and periodontal treatment before 2012 [23]. Dental implants and dentures, which account for the largest expenditures of dental services, were excluded from the dental health insurance coverage [25]. This limited dental coverage created unmet dental needs. According to Korea Health statistics, in 2012, 46.6% of the elderly over the age of 65 had chewing difficulties [26]. They frequently failed to receive essential dental care owing to financial reasons.

Starting in 2012, the NHI reform was implemented to decrease unmet dental needs, particularly among the elderly. Table 1 outlines the gradual dental policy expansion over several years, showing a progressive reduction in patient costs over time. For instance, prior to July 2012, dentures (both full and partial) and implants were not covered by the NHI, requiring all patients to pay 100% of the costs out of pocket. However, beginning on 1 July 2012, individuals aged 75 and above were required to pay only 50% of the cost for full dentures. In the following year, coverage was expanded to include partial dentures for those aged 75 and above, again covering 50% of the costs. Starting in July 2014, 70% of the costs of implants for those aged over 75 were covered. By 1 July 2016, individuals aged 65 and above were only responsible for 30% of the costs across all types of dental treatments, indicating a significant shift toward more affordable dental care for older demographics.

**Table 1:** Expansion of the dental health coverage of the NHI.

	Dentures				Implants	
	Full Dentures		Partial Dentures			
Time of Policy Change	Eligible Age	Co-Payment Rate	Eligible Age	Co-Payment Rate	Eligible Age	Co-Payment Rate
Before	None	100%	None	100%	None	100%
1 July 2012	≥75	50%	None	100%	None	100%
1 July 2013	-	-	≥75	50%	None	100%
1 July 2014	-	-		-	≥75	30%
1 July 2015	≥70	50%	≥70	50%	≥70	30%
1 July 2016	≥65	30%	≥65	30%	≥65	30%

Note: Dashes indicate no change in policy from the previous year. “Before” means until June 30, 2012. Source: Korea National Health Insurance website.

### 2.2. Data Source and Study Population

We examine the impact of expanding dental insurance on dental service utilization and some dental health outcomes among older adults in Korea. The Korea National Health and Nutrition Examination Survey (KNHNES) is a nationally representative cross-sectional survey conducted by the Korea Center for Disease Control (KCDC).

The Korea National Health and Nutrition Examination Survey (KNHANES) was launched in 1998 to provide evidence for the development and evaluation of health policies and programs. It is a nationally representative cross-sectional survey of Korea. In 2007, oral health examinations were introduced for the first time, and oral examinations of the respondents were conducted by dentists every year until 2019. However, dental examinations could not be conducted in 2020 and 2021 due to the COVID-19 pandemic [27]. Our data were obtained from five waves of the survey (waves IV–VIII, corresponding to 2007–2009, 2010–2012, 2013–2015, 2016–2018, and 2019–2021, respectively). However, the seventh wave (2016–2018) provided combined data without distinguishing among individual years. Therefore, we have annual dental information from 2007 to 2019, with that from 2016 to 2018 considered as single-point data.

We restricted our study population to adults aged between 50 and 79 years. The KNHNES dataset top-codes age at 80. Therefore, we restricted our sample to individuals aged 79 and below to maintain a homogeneous group. Our data possess several significant advantages. Each tooth was objectively evaluated by a dentist (for health, dental caries, tooth loss, etc.), who also provided opinions on needed dental treatment. Furthermore, the data provide information on both the upper and lower jaws, including whether individuals had full, partial dentures, fixed bridges, or implants for their upper and lower jaws separately, and indicating whether individuals required full, partial, or fixed bridges. To the best of our knowledge, this is the first study to treat upper and lower jaws separately, thanks to the clean data evaluated by dentists.

### 2.3. Sample Construction

We constructed three samples based on the relevant population.

A denture is a removable replacement for missing teeth and surrounding tissues. Two types of dentures are available—full and partial. Full dentures are used when all the teeth are missing, while partial dentures are used when some natural teeth remain.

Dental implants involve a minor surgical procedure, which requires a healing period afterward. The exact length of the installation from start to finish can vary widely, but it generally takes months [28]. Implants will last longer but also take longer to complete. A dental bridge is attached to the healthy teeth on either side of the missing tooth, which are shaved down and capped with a dental crown. A false tooth is then attached to both caps to fill in the missing tooth [28].

For dental treatment decisions for missing teeth, two factors play a major role: the number of teeth lost and whether the individual wants a removable or fixed option. Individuals need to make separate decisions for their upper jaw and lower jaw. For example, if someone lost all of their teeth in the upper (or lower) jaw, they need full dentures, which are removable appliances. For someone who has lost a set of teeth, but not the entire set of upper (or lower) teeth, they will have two options: partial dentures, which are removable appliances, or fixed bridges or implants, which are permanent options. The bone in the upper jaw is softer compared to that in the lower jaw, which can influence a patient’s choice of treatment options and affect recovery time. This difference, however, was not accounted for in our analysis [29]. Additionally, the KNHNES dataset did not provide the specific timing of the implants, making it impossible to conduct an implant survival analysis. The removable vs. fixed option is an individual preference. A third factor in making a decision might be the cost of each option.

Our dataset contained the following information. A dentist observed the current dental health situations of those with full dentures, partial dentures, a fixed bridge, or implants. The dentist also evaluated whether the patient needed full dentures, partial dentures, or a fixed bridge. We considered that the information about their need reflected the patient’s preference for a removable vs. a fixed option.

We created relevant samples, as follows. The full denture sample comprised the patients who had full dentures or needed full dentures at the jaw level. The partial denture sample comprised patients who had partial dentures or needed partial denture. The implant sample comprised those who had fixed bridge or implants or needed a fixed bridge. Patients who have lost all teeth in the upper or lower jaw might choose implants for all of his/her teeth, but, considering the cost, it is unlikely. Therefore, we did not consider this situation.

Table 2 shows the observations made for each sample. The full denture and partial denture samples comprised 4890 and 12,952 observations, respectively. The implant sample comprised 25,068 observations. We also provided the average price of each dental treatment based on the 2024 information provided by Health Insurance Review & Assessment Service website [30]. The denture prices were similar between the full and partial dentures at around KRW 1.5 million (USD 1154, using the 2023 average exchange rates). This means that before the dental policy expansion, individuals had to pay KRW 1.5 million. With 50% coverage from health insurance, the real price for dentures dropped down to KRW 0.75 million. The price for dental implants was KRW 1.1 million for each. With a 30% out-of-pocket expenditure, the monetary burden for individuals dropped down to KRW 0.3 million.

**Table 2:** Descriptive statistics.

Variables	Full Denture Sample	Partial Denture Sample	Dental Implant Sample
**Average Price** **(30% out-of-pocket)**	KRW 1,510,000 (KRW 460,000)	KRW 1,590,000 (KRW 480,000)	KRW 1,130,000 (KRW 339,000)
**Dependent Variable**			
Has partial denture		0.605 (0.489)	
Has complete denture	0.922 (0.268)		
Has dental implant			0.267 (0.443)
Has severe chewing difficulty	0.236 (0.424)	0.221 (0.415)	0.103 (0.304)
**Independent Variable**			
Male	0.478 (0.500)	0.444 (0.497)	0.430 (0.495)
Age	70.236 (6.625)	66.850 (7.685)	63.660 (7.908)
Has spouse	0.661 (0.473)	0.720 (0.449)	0.795 (0.404)
**Income Group**			
Low	0.550 (0.498)	0.440 (0.496)	0.297 (0.457)
Lower–middle	0.250 (0.433)	0.285 (0.451)	0.276 (0.447)
Upper–middle	0.119 (0.324)	0.161 (0.367)	0.217 (0.412)
High	0.081 (0.273)	0.115 (0.319)	0.210 (0.408)
**Education Group**			
Primary school	0.707 (0.455)	0.611 (0.488)	0.453 (0.498)
Middle school	0.125 (0.331)	0.158 (0.365)	0.181 (0.385)
High school	0.129 (0.334)	0.167 (0.373)	0.240 (0.427)
University	0.040 (0.196)	0.064 (0.245)	0.126 (0.332)
Observations	4890	12,952	25,068

Notes: Standard deviations are presented in parentheses. The first row, out-of-pocket expenditure, is based on the 2024 average prices provided by the Health Insurance Review & Assessment Service website.

### 2.4. Dependent Variables

We used “has full denture”, “has partial denture”, and “has implant” as the dependent variables for the relevant samples. We measured the dental service utilization among the relevant samples.

Another variable measuring oral health status was based on the respondents’ self-reported chewing difficulty. The KNHNES asked the respondents “do you have difficulty or feel uncomfortable when chewing food due to oral problems such as teeth, dentures or gums?”. The individual responses were “no difficulty”, “little difficulty”, “some difficulty”, and “severe difficulty”. We categorized “severe difficulty” as 1 and the rest of the categories as 0.

Table 2 shows that 92.2% of the individuals in the full denture sample had full dentures, whereas 60.5% of those in the partial denture sample had partial dentures. Additionally, 26.7% of the individuals in the sample requiring implants underwent implant placement. The rate of severe chewing difficulty was at least twice as high among the denture sample compared to the implant sample. The average age in the denture sample was slightly higher. The full denture sample showed a lower probability of having a spouse compared to the partial and implant samples, probably due to age [31]. Additionally, the denture sample generally exhibited lower levels of education and income in comparison to the implant samples, which might reflect age differences. The older generation experienced lower education and lower income as well.

### 2.5. Econometric Model

When the treatment is binary, we can denote potential outcomes by $Y_{it}(1)$, the potential outcome under treatment, and $Y_{it}(0)$, the potential outcome without treatment. The (infeasible) individual treatment effect of i at t can be defined as $E\left[Y_{it}(1)-Y_{it}(0)\right]$. Without the loss of generality, the average treatment effect can be written as

(1) $$\tau_{w}:=\sum_{it}w_{it}E\left[Y_{it}(1)-Y_{it}(0)\right]$$

with an adequate definition of weights $w_{it}$. When $E\left[Y_{it}(1)-Y_{it}(0)\right]$ is homogeneous across i for all t, the $\tau_{w}$ is consistently estimable by conventional methods, such as two-way fixed-effect linear regression models that are typically adopted in difference-in-difference (DID) estimation. However, when $E\left[Y_{it}(1)-Y_{it}(0)\right]$ is heterogeneous, the conventional DID estimator may estimate $\sum_{it}w_{it}E\left[Y_{it}(1)-Y_{it}(0)\right]$ with rather strange $w_{it},$ such as negative weights, especially when treatment timings are different [24,32,33,34,35]. A few new methods have been developed that take this problem into account [24,32,35], out of which we used the imputation-based method proposed by Borusyak et al. (2024) [24].

The intuition behind their method is rather simple. Suppose $E\left[Y_{it}\left(0\right)|X_{it}\right]=X_{it}^{\prime }\beta$ and β is consistently estimable using individuals’ untreated data (not-yet-treated observations for treated individuals and all observations for never-treated individuals; we used $it\in \Omega_{0}$ for such observations). Then, for individuals’ treated data (treated individuals’ observations after treatment timings; we used $it\in \Omega_{1}$ for such observations), ${\hat{Y}}_{it}\left(0\right):=X_{it}^{\prime }\hat{\beta }$ can be computed and used as an “imputed value” for $E\left[Y_{it}\left(0\right)|X_{it}\right]$. Therefore, a natural estimator for $\tau_{w}$ is as follows:

(2) $${\hat{\tau }}_{w}:=\sum_{it\in \Omega_{1}}w_{it}\left(Y_{it}-{\hat{Y}}_{it}\left(0\right)\right)$$

since $Y_{it}=Y_{it}(1)$ for $it\in \Omega_{1}$. Simply put, we performed linear regression with the untreated data, projected the results to the treated data, and computed the weighted averages of differences between the real outcome variable data and the projected outcome variable values, putting aside the computation of standard errors. We used sample weights in $\Omega_{1}$ as the weight.

Borusyak et al. (2024) allowed for a much more general specification for $E\left[Y_{it}\left(0\right)|X_{it}\right]$ (Assumption 1 in [24]) and proved ${\hat{\tau }}_{w}$ is unbiased (Theorem 2 in [24]) and consistent (Proposition 7 in [24]) under some assumptions, such as parallel trends (Assumption 1 in [24]), the correct specification of $E\left[Y_{it}\left(0\right)|X_{it}\right]$, and no-anticipation effects (Assumption 2 in [24]), among others. They also established the asymptotic normality of ${\hat{\tau }}_{w}$ (Proposition 8 in [24]). Parallel trends in the imputation-based model proposed by Borusyak et al. (2024) can be tested by running separate regression analyses on non-treated observations [24]. We examined the pre-trend over five periods, testing the null hypothesis that the coefficients from Pre1 to Pre5 are jointly equal to zero. The resulting *p*-values for Models 1 to 5 were 0.30, 0.82, 0.52, 0.11, and 0.52, respectively.

Our baseline econometric model for imputation-based estimation proposed by Borusyak et al. (2024) is as follows [24]:

(3) $$y_{i}\left(0\right)=\beta_{0}+\beta_{1}X_{i}+\sum_{l=2}^{T}\delta_{w}\cdot 1\left(C_{i}=C^{l}\right)+\sum_{a=51}^{79}\gamma_{a}\cdot 1\left({age}_{i}=a\right)+\varepsilon_{i}$$

where $wave=C_{i}\;\in \left\{c^{1},\;c^{2},\;\cdots c^{T0},\;c^{T1}\right\}$. $C_{i}$ is the wave of KNHNES. $c^{1},\;c^{2},\;\cdots c^{T0}$ represent the waves before policy intervention, and $c^{T1}$ represents the wave after policy intervention. Our data consist of repeated cross-sections, where age serves as the time variable. $\beta_{0}$, the constant term, reflects the expected value of the outcome when all of the predictor variables in the model are equal to 0, which means age 50 (reference group) and the initial wave. We included age-specific dummies ($\gamma_{a})$ and wave-specific dummies ($\delta_{t})$ to control for nonlinear age and time-specific heterogeneity, respectively. The covariates, $X_{it}$, in our estimation, are as follows: male, income level (low/lower–middle/upper–middle/high, with low as the reference group), educational level (primary school/middle school/high school/university, with primary school as the reference group), and having a spouse.

Once the model was estimated, we defined the estimated non-treated outcome as shown in Equation (4) and used it to compute the “imputed” potential outcome.

(4) $$\hat{y_{i}\left(0\right)}=\hat{\beta_{0}}+\hat{\beta_{1}}X_{i}+\sum_{l=2}^{T}\hat{\delta_{w}}\cdot 1\left(C_{i}=C^{l}\right)+\sum_{a=51}^{79}\hat{\gamma_{a}}\cdot 1\left({age}_{i}=a\right)$$

We estimated two different models for full dentures based on the different policy interventions listed in Table 1. Model 1 utilized the 2012 policy change that reduced the out-of-pocket expenditure for full dentures for those aged 75 and above by 50%. The available survey data for Model 1 were from 2007 to 2014, predating another policy change in 2015 (those aged 70 and over received 50%). Model 2 utilized the policy modification from July 2016, involving a reduction in the ages covered to 65 and over, coupled with a decrease in the out-of-pocket expenditure to 30%. We used data from 2007 to 2011 for before the policy implementation and those from 2016 to 2019 for after the policy implementation. We excluded the data from 2012 to 2015 due to another policy change in 2012 (those aged 75 and over received 50%).

For the partial denture sample, we analyzed Model 3 and Model 4 using the policy changes in 2013 and 2016 as the interventions, respectively. Model 3 was based on the 2013 policy change that reduced the out-of-pocket cost of partial dentures for those aged 75 and above by 50%. The available survey data for Model 3 spanned from 2007 to 2014, predating another policy change in 2015 (those aged 70 and over received 50%). Model 4 was based on the 2016 policy change that reduced the out-of-pocket cost of partial dentures for those aged 65 and above by 70%. We used the data from 2007 to 2012 for before the policy implementation and those from 2016 to 2019 for after the policy implementation. We excluded the data from 2013 to 2015 due to the presence of another policy change in 2013 (those aged 75 and over received 50%).

For the implant sample, we utilized Model 5. Given the policy adjustments concerning dental implants every year since 2014, our estimation focused on the policy treatment effect for the final adjustment year, that is, 2016. This involved a 70% reduction in out-of-pocket expenditures for dental implants for adults aged 65 and over. The available survey data spanned from 2007 to 2013 for before the policy intervention and from 2016 to 2019 for after the policy intervention.

## 3. Results

The imputation estimates, derived from the estimation of the equations outlined in Section 4, are presented in Table 3, Table 4, and Table 5, respectively. Within each table, the first row, labeled ${\hat{\tau }}_{w},$ shows the average policy intervention effects on the dependent variables, like “has full dentures”, in Table 3.

### 3.1. Dental Service Utilization

Table 3 shows the impact of dental insurance coverage expansion on the use of full dentures. The first column in Table 3 presents the results for Model 1, followed by Model 2 in the second column. The imputation estimates presented in the first row indicate that the expansion of the insurance coverage for full dentures did not result in significant changes in full denture utilization. The coefficient in Model 2 means that the policy intervention for full dentures resulted in a 2.4% increase, which is only 2.6% of the sample mean (=2.4/90.7). This insignificant result is not surprising. Once a patient loses all their teeth in the upper or lower jaw, they need full dentures immediately to maintain normal speech and provide occlusal support for adequate chewing [36]. For edentulous patients, full dentures have long been the only prosthodontic treatment option [37]. The United States showed that 90% of individuals requiring full dentures have made use of them [38].

**Table 3:** Effects of dental insurance coverage expansion on full denture service utilization.

	Has Full Dentures	
	Model 1	Model 2
	Policy intervention: Since 2012, for individuals aged 75 and above. Pre-intervention: 2007–2011 Post-intervention: 2013–2014 OOP expenditure: 100 to 50%	Policy intervention: Since 2016, for individuals aged 65 and above. Pre-intervention: 2007–2011 Post-intervention: 2016–2019 OOP expenditure: 100 to 30%
${\hat{\tau }}_{w}(treatment\;effect)$	−0.0290	0.0244
	(0.0308)	(0.0363)
Male	−0.0552 ***	−0.0555 ***
	(0.0164)	(0.0174)
Spouse	0.0367 **	0.0272
	(0.0163)	(0.0175)
Middle school	0.0451 **	0.0554 **
	(0.0209)	(0.0223)
High school	0.00431	0.0218
	(0.0243)	(0.0278)
University	0.00443	0.000421
	(0.0343)	(0.0389)
Income (low–middle)	−0.00740	0.00699
	(0.0174)	(0.0185)
Income (upper–middle)	0.00240	0.00664
	(0.0230)	(0.0247)
Income (high)	0.0566 ***	0.0536 **
	(0.0203)	(0.0227)
		
Observations	3091	3466
Dependent variable mean	0.912	0.907

Note: Age-specific dummies and wave-specific dummies were included to control for nonlinear age and time-specific heterogeneity, respectively. Standard errors are presented in parentheses. ** and *** indicate significance at the 5% and 1% levels, respectively.

Table 4 shows the impact of dental insurance coverage expansion on the use of partial dentures. Model 4 shows that the expansion of insurance coverage for partial dentures increased the probability of wearing partial dentures among people aged 65 and above by 8.96 percentage points. This is a statistically significant effect at 13.5% (=0.089/0.660) and reflects a big impact on magnitude. The price elasticity can be calculated as the utilization change in percent (0.135) divided by the price change in percent. Out-of-pocket expenses decreased from 100% to 30%, representing a 70% drop in price. Therefore, the price elasticity of partial dentures was −0.19 (0.135/−0.7). Model 3 did not show a statistically significant impact, and its coefficient was considerably smaller compared to Model 4. There are two possible explanations for why Model 3 did not show statistically significant results but Model 4 did. First, we had only one year of data from after the policy implementation, which was 2014. Second, the coverage rate was smaller than in Model 4.

**Table 4:** Effects of dental insurance coverage expansion on partial denture service utilization.

	Has Partial Dentures	
	Model 3	Model 4
	Policy intervention: Since 2013, for individuals aged 75 and above. Pre-intervention: 2007–2012 Post-intervention: 2014 OOP expenditure: 100 to 50%	Policy intervention: Since 2016, for individuals aged 65 and above. Pre-intervention: 2007–2012 Post-intervention: 2016–2019 OOP expenditure: 100 to 30%
${\hat{\tau }}_{w}(treatment\;effect)$	0.0299	0.0896 ***
	(0.0462)	(0.0266)
Male	−0.0758 ***	−0.0727 ***
	(0.0142)	(0.0139)
Spouse	0.0261 *	0.0272 *
	(0.0155)	(0.0151)
Middle school	−0.00928	−0.0207
	(0.0189)	(0.0185)
High school	−0.0291	−0.0276
	(0.0194)	(0.0189)
University	−0.00286	−0.0104
	(0.0283)	(0.0275)
Income (low–middle)	0.0142	0.0160
	(0.0154)	(0.0154)
Income (upper–middle)	0.0429 **	0.0421 **
	(0.0193)	(0.0190)
Income (high)	0.0465 **	0.0499 **
	(0.0209)	(0.0206)
		
Observations	8427	10,172
Dependent variable mean	0.667	0.660

Notes: Age-specific dummies and wave-specific dummies were included to control for nonlinear age and time-specific heterogeneity, respectively. Standard errors are presented in parentheses. *, ** and *** indicate significance at the 10%, 5% and 1% levels, respectively.

Table 5 shows that the implant insurance coverage expansion significantly increased the probability of having implants among people aged 65 and above by 6.9 percentage points. The treatment effect of this policy was a 60.5% increase in the sample mean (0.069/0.114). The biggest treatment effect related to dental implants shows that the provision of dental implants to replace missing teeth for older adults made this a popular treatment option [39,40]. The price elasticity of implants was −0.86 (0.605/−0.7).

**Table 5:** Effects of dental insurance coverage expansion on dental implant service utilization.

	Has Dental Implants
	Model 5
	Policy intervention: Since 2016, for individuals aged 65 and above. Pre-intervention: 2007–2013 Post-intervention: 2016–2019 OOP expenditure: 100 to 30%
${\hat{\tau }}_{w}(treatment\;effect)$	0.0690 ***
	(0.0150)
Male	−0.0206 ***
	(0.00733)
Spouse	0.0269 ***
	(0.00829)
Middle school	0.0321 ***
	(0.00926)
High school	0.0867 ***
	(0.00981)
University	0.148 ***
	(0.0134)
Income (low–middle)	0.0195 **
	(0.00819)
Income (upper–middle)	0.0613 ***
	(0.00973)
Income (high)	0.108 ***
	(0.0108)
Observations	21,332
Dependent variable mean	0.114

Note: Age-specific dummies and wave-specific dummies were included to control for nonlinear age and time-specific heterogeneity, respectively. Standard errors are presented in parentheses. ** and *** indicate significance at the 5% and 1% levels, respectively.

### 3.2. Chewing Difficulty

Using the imputation-based method, this section presents estimates of the impact of having partial dentures or dental implants following policy intervention on alleviating severe chewing difficulty. $Y_{i}$ in Model 1 is a binary variable that indicates whether individual i has severe chewing difficulty.

According to the imputation estimates, as the insurance coverage expansion policy did not notably increase the utilization of full dentures and partial dentures among the population aged over 75, we only report the models showing statistically significant results in the previous section.

Table 6 presents the impact of policy intervention on severe chewing difficulty. Columns 1 and 2 report the estimation results for the partial denture policy and dental implant policy, respectively, on severe chewing difficulty. In Column 1, it can be observed that lowering the price of partial dentures for those aged 65 and over was associated with a significant alleviation of severe chewing difficulty, as indicated by a reduction of 6.07 percentage points. Considering the sample mean of 0.238, this corresponds to a decrease in severe chewing difficulty of 23.3% (=0.0607/0.260). In Column 2, it can be seen that lowering the price of dental implants had a significant effect on reducing the probability of experiencing severe chewing difficulty by 2.13 percentage points. Considering the sample mean of 0.124, this translates to a decrease in severe chewing difficulty by 13.0% (=0.021/0.161). The out-of-pocket price with both policy interventions went from 100% to 30%. The price change of partial dentures showed a bigger impact on severe chewing difficulty compared to that of dental implants. This may be because the proportion of people with severe chewing difficulties was smaller among those who need dental implants. It was found that 13% of individuals needing dental implants experienced severe chewing difficulty, compared to 29% among those who needed partial dentures. Therefore, wearing partial dentures had a more significant marginal improvement effect on severe chewing problems.

### 3.3. Sensitivity Analysis

This section presents a sensitivity analysis of the data in Table 3, Table 4, Table 5 and Table 6, which was performed by varying the age ranges included in this study. The original sample, following Kim et al. (2023), defined the study population as individuals aged 50 to 79 [41]. We performed three sensitivity analyses: (1) restricting the study population to those aged 55 to 79, (2) restricting it to those aged 60 to 79, and (3) including populations 10 years younger and older than the eligible age cutoff. It is important to note that individuals use dental care services when needed, rather than when a policy is implemented. Therefore, the population that utilizes these services can vary in age within the eligible range. For instance, a 67-year-old individual might need an implant in 2018, even though they have been eligible for dental insurance coverage since 2016. Due to this pattern, our sensitivity analysis changed only for age groups younger than the eligible age range. The decision to use services in 2018 at age 67, rather than in 2016 at age 65, reflects this behavior. The results of these analyses are presented in Appendix A, Table A1 and Table A2. While the magnitude of the effects changed slightly, the overall results remain robust.

## 4. Discussion

Globally, there is a widespread issue of high rates of tooth loss among the elderly, significantly impacting their basic functions, such as chewing and speech. Tooth loss is recognized by the World Health Organization as one of the major threats to oral health, alongside dental caries, periodontal diseases, and oral cancer [2]. According to estimates from the Centers for Disease Control and Prevention (CDC) in the United States, the prevalence of complete tooth loss among adults aged 65 and over is 12.9% and increases with age. Among those aged 65 to 69, 8.9% of Americans are completely edentulous; this proportion rises to 10.6% in the 70 to 74 age group, and reaches as high as 17.8% in those aged 75 and above [42]. Good oral health is an important part of healthy aging, yet oral health has not received sufficient attention in most countries and has even been considered a low-priority issue at times.

While some countries have implemented policies aimed at improving the oral health of the elderly population, these policies mainly emphasize the importance of oral hygiene for older adults or provide educational campaigns highlighting the relationship between oral health and overall health for both elderly individuals and healthcare professionals [10]. Due to the high cost of dental services, only a few countries have enacted policies to alleviate the financial burden of dental services for the elderly. Korea gradually implemented dental denture and implant coverage in 2012 for the elderly, which provided us with a great opportunity to examine the policy effects on utilization and chewing ability. This study evaluated the impact of health insurance policy on dental service utilization and oral health outcomes in Korea.

We used the Korea National Health and Nutrition Examination Survey (KNHNES), a nationally representative cross-sectional survey of Korea, to measure the utilization of dental services as assessed by a dentist and oral health outcomes and reported by survey respondents. We estimated different models for denture/dental implant utilization, as the time of insurance coverage expansion for full dentures, partial dentures, and dental implants differed.

The results based on the imputation analysis show that the policy increased the proportions of partial dentures and dental implants used by adults aged 65 and above by 13.5% and 60.5%, respectively, but there was no significant change in the use of full dentures. The use of partial dentures and dental implants significantly reduced chewing difficulty by 23.3% and 13.0%, respectively.

The results of our study indicate that expanding dental insurance coverage for individuals aged 75 and over did not show any statistically significant effects on dental service utilization, whereas significant effects were observed for those aged 65 and over. This discrepancy can be partially attributed to the life expectancy in Korea, which is approximately 83 years [43]. As people age, particularly beyond 75, the feasibility and efficacy of dental care interventions may diminish due to declining overall health and increased frailty. Consequently, the benefits of such care may not be as pronounced in this older cohort. This finding suggests that a more effective age cutoff for the expansion of dental insurance coverage is 65 and over, as it aligns better with the period when dental care can still significantly enhance one’s quality of life and chewing ability. Prioritizing dental care for this age group ensures that interventions are both practical and beneficial, addressing the critical dental health needs before individuals become too old for such treatments to be viable or effective.

In our analysis, we calculated the price elasticity of demand for statistically significant results, finding an elasticity of −0.19 for partial dentures and −0.86 for dental implants. These results are similar to those of Manning et al.’s (1987) randomized control trial, which reported a dental care elasticity of −0.39, and Eichner’s (1998) estimates ranging from −0.62 to −0.75 based on out-of-pocket costs [44,45]. Our findings indicate that dental implants are more elastic compared to partial dentures. This greater elasticity can be attributed to the higher cost of dental implants, especially considering the price incurred for each tooth lost. Consequently, the total cost for individuals with multiple missing teeth can be substantially higher for implants than for partial dentures. This price sensitivity highlights the importance of affordability in increasing the utilization of dental implants among the elderly, emphasizing the need for targeted financial support to make such treatments more accessible. Another possible explanation for the difference in elasticity is the variation in marginal returns. When a patient requires partial dentures, the marginal returns from obtaining them can be higher compared to when a patient requires only dental implants. This explanation is also consistent with the observation that there was no statistically significant change in the demand for full dentures, despite a decrease in the out-of-pocket expenditures. This is likely because when a patient needs full dentures, they are more likely to obtain them regardless of the cost, as life can be very challenging without them.

Our study might have limited implications for other countries since their general nutrition, health conditions, and the medical environment can play roles in an individual’s decision.

## 5. Conclusions

This study found that expanding dental insurance coverage could increase the use of partial dentures by 13.5% and dental implants by 60.53% among individuals aged 65 and over. These changes would result in reductions in the incidence of severe chewing difficulty by 23.3% and 13.0%, respectively. However, the expansion of coverage did not significantly impact the use of full dentures. We calculated the price elasticity rates of demand for partial dentures and dental implants to be −0.19 and −0.86, respectively, indicating that the high cost of dental implants makes them more price-elastic compared to partial dentures. The government could provide targeted financial support by reducing the cost of dental implants or increasing the reimbursement rates, making dental implant treatment more accessible.

## Figures and Tables

**Table 6:** Effects of having dental attachments on severe chewing difficulty.

	Has Severe Chewing Difficulty	
	(1)	(2)
	Partial Denture Policy Policy intervention: Since 2016, for individuals aged 65 and above. Pre-intervention: 2007–2012 Post-intervention: 2016–2019	Dental Implant Policy Policy intervention: Since 2016, for individuals aged 65 and above. Pre-intervention: 2007–2013 Post-intervention: 2016–2019
${\hat{\tau }}_{w}(treatment\;effect)$	−0.0607 ***	−0.0213 **
	(0.0232)	(0.0102)
Male	0.0169	0.0237 ***
	(0.0126)	(0.0061)
Spouse	−0.0572 ***	−0.0323 ***
	(0.0145)	(0.0085)
Middle school	−0.0443 ***	−0.0450 ***
	(0.0149)	(0.0070)
High school	−0.0796 ***	−0.0593 ***
	(0.0157)	(0.0072)
University	−0.0845 ***	−0.0830 ***
	(0.0229)	(0.0067)
Income (low–middle)	−0.0401***	−0.0479 ***
	(0.0143)	(0.0088)
Income (upper–middle)	−0.0382 **	−0.0527 ***
	(0.0171)	(0.0091)
Income (high)	−0.0845 ***	−0.0589 ***
	(0.0229)	(0.0090)
Observations	10,172	21,332
Dependent variable mean	0.260	0.161

Notes: Age-specific dummies and wave-specific dummies were included to control for nonlinear age and time-specific heterogeneity, respectively. Standard errors are presented in parentheses. **, and *** indicate significance at the 5%, and 1% levels, respectively.

## Data Availability

Raw data of this article are available upon request to the corresponding author.

## Appendix A

**Table A1:** Sensitivity analysis for denture service utilization.

	Has Full Dentures		Has Partial Dentures		Has Dental Implants
	Model 1	Model 2	Model 3	Model 4	Model 5
Restricted study population to adults aged between 50 and 79 years.					
${\hat{\tau }}_{w}$	−0.0290 (0.0308)	0.0244 (0.0363)	0.0299 (0.0462)	0.0896 *** (0.0266)	0.0690 *** (0.0150)
Restricted study population to adults aged between 55 and 79 years.					
${\hat{\tau }}_{w}$	−0.0271 (0.0308)	0.0273 (0.0365)	0.0241 (0.0465)	0.0839 *** (0.0286)	0.0581 *** (0.0163)
Restricted study population to adults aged between 60 and 79 years.					
${\hat{\tau }}_{w}$	−0.0182 (0.0311)	0.0448 (0.0409)	0.0116 (0.0476)	0.0325 (0.0349)	0.0417 ** (0.0202)
Restricted study population to adults 10 years younger or older than the eligible age cutoff.					
${\hat{\tau }}_{w}$	−0.0163 (0.0324)	0.0148 (0.0371)	0.0222 (0.0494)	0.0731 ** (0.0300)	0.0544 *** (0.0172)

Notes: Gender, marital status, education level, and income were included as independent variables. Age-specific dummies and wave-specific dummies were included to control for nonlinear age and time-specific heterogeneity, respectively. Standard errors are presented in parentheses. **, and *** indicate significance at the 5%, and 1% levels, respectively.

**Table A2:** Sensitivity analysis for severe chewing difficulty.

	Has Severe Chewing Difficulty	
	Partial Denture Policy	Dental Implant Policy
Restricted study population to adults aged between 50 and 79 years.		
${\hat{\tau }}_{w}$	−0.0607 *** (0.0232)	−0.0213 ** (0.0102)
Restricted study population to adults aged between 55 and 79 years.		
${\hat{\tau }}_{w}$	−0.0964 *** (0.0250)	−0.0529 *** (−0.0110)
Restricted study population to adults aged between 60 and 79 years.		
${\hat{\tau }}_{w}$	−0.0824 *** (−0.0307)	−0.0478 *** (−0.0133)
Restricted study population to adults 10 years younger or older than the eligible age cutoff.		
${\hat{\tau }}_{w}$	−0.0862 *** (−0.0261)	−0.0461 *** (−0.0114)

Notes: See notes in Table A1.
